# Acceptance of Combined Coronary CT Angiography and Myocardial CT Perfusion versus Conventional Coronary Angiography in Patients with Coronary Stents—Intraindividual Comparison

**DOI:** 10.1371/journal.pone.0136737

**Published:** 2015-09-01

**Authors:** Matthias Rief, Sarah Feger, Peter Martus, Michael Laule, Marc Dewey, Eva Schönenberger

**Affiliations:** 1 Department of Radiology, Charité, Medical School, Berlin, Germany; 2 Institute for Clinical Epidemiology and Applied Biostatistics, Eberhard Karls University Tübingen, Germany; 3 Department of Cardiology, Charité, Medical School, Berlin, Germany; 4 Department of Medicine, Medical School Hannover, Hannover, Germany; University of Bologna, ITALY

## Abstract

**Objectives:**

To evaluate how well patients with coronary stents accept combined coronary computed tomography angiography (CTA) and myocardial CT perfusion (CTP) compared with conventional coronary angiography (CCA).

**Background:**

While combined CTA and CTP may improve diagnostic accuracy compared with CTA alone, patient acceptance of CTA/CTP remains to be defined.

**Methods:**

A total of 90 patients with coronary stents prospectively underwent CTA/CTP (both with contrast agent, CTP with adenosine) and CCA as part of the CARS-320 study. In this group, an intraindividual comparison of patient acceptance of CTA, CTP, and CCA was performed.

**Results:**

CTP was experienced to be significantly more painful than CTA (p<0.001) and was associated with a higher frequency of dyspnea (p<0.001). Comparison of CTA/CTP with CCA revealed no significant differences in terms of pain (p = 0.141) and comfort (p = 0.377). Concern before CTA/CTP and CCA and overall satisfaction were likewise not significantly different (p = 0.097 and p = 0.123, respectively). Nevertheless, about two thirds (n = 60, 68%) preferred CTA/CTP to CCA (p<0.001). Moreover, patients felt less helpless during CTA/CTP than during CCA (p = 0.026). Lack of invasiveness and absence of pain were the most frequently mentioned advantages of CTA/CTP over CCA in our patient population.

**Conclusions:**

CCA and combined CTA/CTP are equally well accepted by patients; however, more patients prefer CTA/CTP. CTP was associated with more intense pain than CTA and more frequently caused dyspnea than CTA alone.

**Trial Registration:**

ClinicalTrials.gov NCT00967876

## Introduction

Percutaneous coronary stent implantation is a widely used treatment option besides medical treatment in patients with stable coronary heart disease [1]. Relevant complications of stenting include coronary in-stent restenosis [2] and neoatherosclerosis [3]. In general, stress tests are recommended to detect possible in-stent restenosis in symptomatic patients with coronary stents. If the clinical presentation or the stress test points to in-stent stenosis, conventional coronary angiography (CCA) is recommended [4]. While coronary computed tomography angiography (CTA) is an established diagnostic procedure, along with CCA, for evaluating the native coronary arteries [5], coronary stents often produce considerable artifacts that may limit the diagnostic evaluability of stented segments [6, 7].

Some recent studies have investigated whether the diagnostic accuracy of cardiac CT can be improved by combining CTA with myocardial CT perfusion (CTP) imaging [8–10]. Intravenous contrast medium injection is required for both CTA and CTP. CTP additionally involves pharmacologically induced stress, typically with administration of adenosine [11]. The technical feasibility of combined CTA/CTP has been established in earlier studies, while it is still unclear how well this new cardiac CT test is accepted by patients. Patient acceptance is a relevant clinical aspect to be considered when introducing a new diagnostic tool as patient integration in decision making has been shown to increase compliance and the clinical outcome [12]. We therefore assessed patients’ acceptance of CTA/CTP and CCA in an ancillary study of the prospective intention-to-diagnose CARS-320 study [13].

## Methods

### Ethics statement

This clinical trial has been registered (www.clinicaltrials.gov; Study NCT00967876; August 27, 2009) and all patients gave written informed consent for the study. We performed the study based on the Declaration of Helsinki and in accordance with the TREND guidelines [14] that correspond to the CONSORT guidelines for randomized trials. The TREND checklist (**S1 TREND Checklist**) is attached as supporting material. The study protocol was approved by the Charité IRB (EA1/133/08) and the Federal Office for Radiation Protection (BfS Z5-22462/2-2008-057) and is available as supporting material (**S1 Protocol**). The authors confirm that all ongoing and related trials for this intervention are registered.

### Study design

Here we present a subanalysis of patients’ perspectives from the intention-to-diagnose *C* *oronary* *Ar* *tery* *S* *tent Evaluation with* *320* *-row CT* study, the main diagnostic accuracy results of which have been recently published and, due to that, are not part of this analysis [13] (CARS-320). All patients underwent combined CTA and CTP with adenosine administration, followed by CCA [13]. Patient enrolment was between April 2, 2009 and November 23, 2011. All patients and stents were included in the CARS-320 study (the slight delay in trial registration is due to administrative reasons; see www.clinicaltrials.gov for further details). Clinical follow-up was performed 6 months, 12 months and 24 months after CT examination (last follow-up in 11/2013) without undergoing coronary CT or CCA. Patient acceptance of the examinations was assessed by means of a validated questionnaire, comparing combined CTA/CTP versus CCA that has already been published in PLoS ONE [15]. Moreover, CTA was compared with CTP following adenosine provocation. Physicians performed the patient information about the scheduled examinations and all patients gave written informed consent for the analysis of patient acceptance.

### Study population and CT protocol

**Fig 1:**
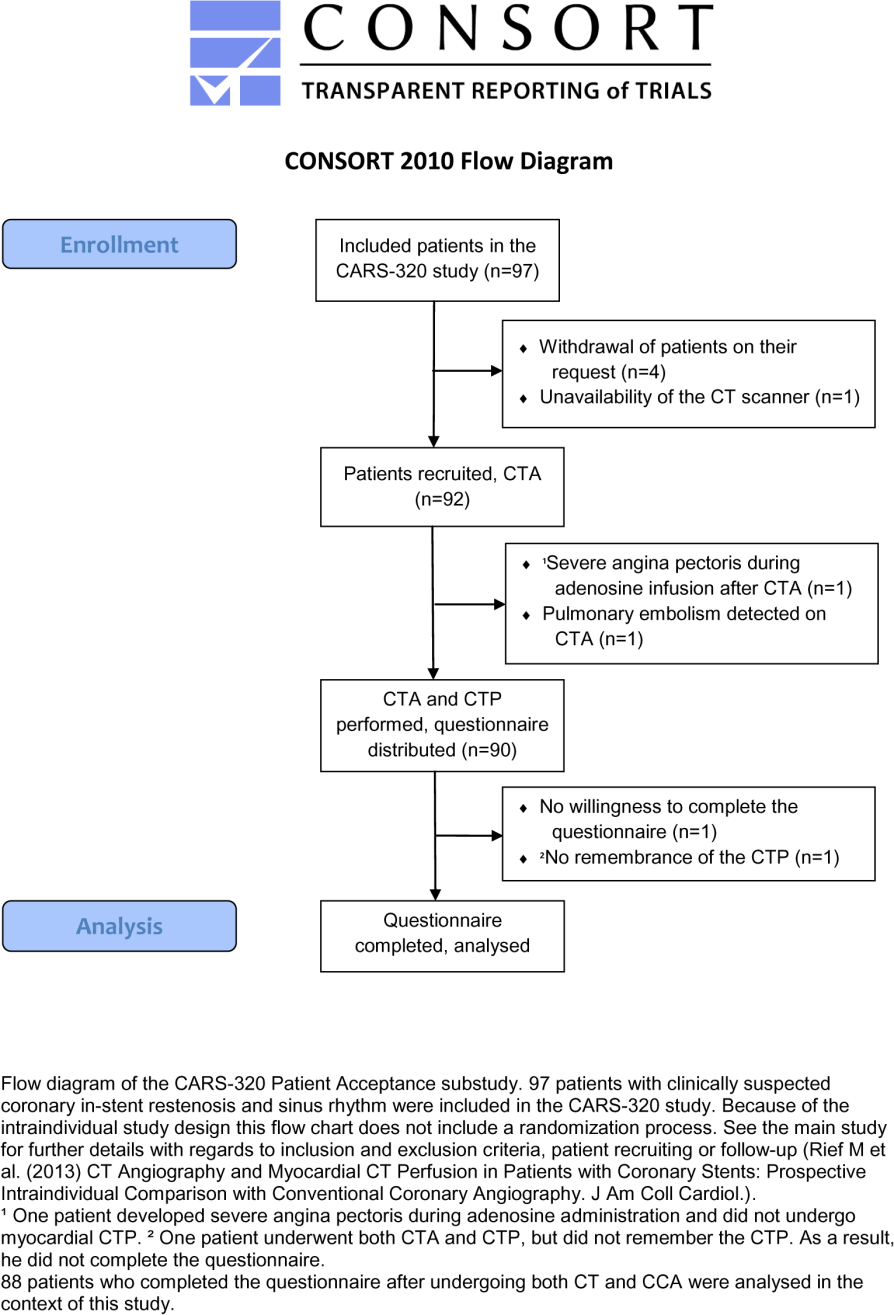
CONSORT Flow Diagram.

Ninety patients with suspected in-stent restenosis and an indication for CCA [13] were included and asked to complete a questionnaire on patient acceptance. Eighty-eight patients answered all questions. **Fig 1** shows the CONSORT Flow Diagram of this study. The inclusion and exclusion criteria of the study, the CT protocol, and the clinical indications of each examination have been described in detail before [13]. In brief, the patients first underwent CTA, followed by CTP with adenosine administration. The CT examinations were performed on a CT scanner with a 320-row detector (Aquilion One, Toshiba, Tokio, Japan) [16]. In 62 patients (82%) beta-blockers were administered orally 1h before CTA (atenolol, Tenormin, Astra-Zeneca). An additional intravenous beta-blockade (esmolol, Brevibloc, Baxter) was performed in 15 patients (17%). Contrast medium (Imeron 400, Bracco Imaging, Milano, Italy) was injected into the right cubital vein using a standardized protocol. CTP with adenosine administration was performed after an interval of at least 20 min. Adenosine (Adenosin Life Medical, Carinopharm, Elze, Germany, 140μg/kg/min) was administered through the line in the left antecubital vein using a perfusor system. Approximately 4.5 min after initiation of adenosine administration, a second contrast bolus was administered and the scan was started.

### Conventional Coronary Angiography

CCA was performed after CTA/CTP using the routine clinical protocol and angiographic projections were standardised [17]. The femoral artery was punctured after local anesthesia with approx. 150–200 mg lidocaine (Lidoject, Hexal AG, Holzkirchen, Germany). A nonionic, iodinated contrast agent (iobitridol, Xenetix 350, Guerbet, Villepinte, France) was used. Fractional flow reserve (FFR) was measured in a total of 8 patients. Adenosine (140 μg/kg/min; Adenosin Life Medical, Carinopharm GmbH, Gronau/Leine, Germany) was infused intra-arterially via the cardiac catheter for 3 to 5 min, at the cardiologist’s discretion to identify culprit lesions [18]. After the examination, a suture closure device was used in 66 cases (AngioSeal, St. Jude Medical, Minnesota, USA; Starclose and Perclose, Abbott Laboratories, Illinois, USA), and 4–6 h of bed rest were ordered. The compression bandage was removed after 2–3 h. In the 22 cases where manual compression was used, 12 h of bed rest were required, and the compression bandage was removed after 6 h.

### Questionnaire

The aim of the questionnaire and its structure (**S1 File**) were explained in detail to the patients before the examinations took place to make sure that they would answer the questions adequately. The information provided included a detailed explanation of the difference between CTA and CTP. Twenty-four hours after the end of CCA, the patients completed the questionnaire, describing how they felt before and during CTA/CTP and CCA and reporting what they subjectively perceived to be relevant advantages and disadvantages of each test. The questions were identical for CTA/CTP and CCA. Patients were asked to rate preparation for the test and the information they were given, their comfort during the examination, and their overall satisfaction with each test using a 5-point scale ranging from “very good” to “very poor”. Using the same scale ranging from “no concern” to “very intense” patients rated their degree of concern before the examination. The occurrence of undesired effects and the willingness to undergo the examination again were reported using a nominal scale and questions to be answered by “yes”, “no”, or “don’t know”. In addition, patients were asked which procedure they preferred. Open-ended questions allowed patients to give reasons for their concern, to report the complications they suffered, and to describe the pros and cons of the two procedures. Subjective pain was rated using a nonmarked 100 mm horizontal visual analogue scale [19]; in addition, patients were asked to mark in different colors the share of pain they attributed to CTA and CTP. A separate set of questions pertained to a direct comparison of CTA and CTP with adenosine. Here, patients were asked to describe their concern, dyspnea, pain, and willingness to undergo the test again. Concern was rated using the same 5-point scale as described above. In addition, patients could give reasons for their concern, and rated subjective pain on a VAS. Yes/no questions were used to ask about dyspnea and chest pain.

### Statistical analysis

Unless otherwise stated, data are presented as mean ± standard deviation. Statistical significance was assumed at p ≤0.05. Patient satisfaction with the different procedures was compared using the signed-rank test. Patient preferences were analyzed using the chi-square or Fisher exact test [15]. VAS-based subjective pain intensity ratings were analyzed using the Wilcoxon test for paired samples. Subgroup analyses (e.g., patients with/without revascularization) were performed using the Mann-Whitney U-test. Beforehand, normal distribution was refuted using the Kolmogorov-Smirnov test. All statistical analyses were performed using SPSS version 20.0 (SPSS Inc.Chicago, IL, USA).

## Results

According to the protocol of the CARS-320 study all patients received CTA followed by CTP, which finally increased the per-patient diagnostic accuracy in the main diagnostic accuracy analysis [13]. Afterwards each patient underwent the clinically indicated CCA as the reference method independent of the results of CTA and CTP (intention-to-diagnose design of CARS-320). For the present ancillary analysis 88 of the 90 patients (98% response rate) who underwent CTA/CTP and CCA as part of CARS-320 answered all items of the patients`preference questionnaire (**S1 Table**). The two patients who did not complete the questionnaire were a 65-year-old man (two coronary stents in the left anterior descending coronary artery, implantation of two stents into the right coronary artery during study procedure) and a 71-year-old man (one coronary stent in the left circumflex coronary artery, no stent implantation during study procedure). None of these two patients had in-stent restenosis. The 88 patients who completed the questionnaires had a mean age of 64 years (**Table 1**), and there was a male-to-female ratio of 4:1. Before inclusion into the study, the patients had an average number of 2.5 coronary stents. CTA/CTP was performed on average 6 days after study inclusion, and 80% of the study patients underwent CCA on the same day as CT (mean delay of 17h 35min; range, 77 min to 12 days 23h 42min). All patients first underwent CTA/CTP. The subsequent CCA was clinically indicated in all cases. 42 patients (48%) underwent percutaneous intervention during the CCA, and a FFR was performed in 8 patients (9%).

**Table 1:** Characteristics of the 88 Included Patients Who Completed the Analysis.

Feature			
Age		63.9	±9.9 years
Sex	Female	17	(19%)
	Male	71	(81%)
Abdominal circumference		101.3	±10.2 cm
Height		172.2	±7.8 cm
Weight		82.1	±12.2 kg
BMI		27.7	±3.9
Systolic blood pressure		128.7	±16.0 mmHg
Diastolic blood pressure		79.7	±8.3 mmHg
Cardiac insufficiency *^1^	I	9	(10%)
	II	58	(66%)
	III	19	(22%)
	IIII	2	(2%)
Myocardial infarction *^2^		40	(45%)
Number of stents per patient		2.5	±1.8
Cardiac pacemaker		1	(1%)
ICD		3	(3%)
Hypertension		73	(83%)
Hyperlipidemia		74	(84%)
Smoking		17	(19%)
Diabetes mellitus		23	(26%)
Interval between enrolment and CT *^3^		0,1,1,1,42	days
Interval between CT and CCA *^3^		0,0,0,0,12	days

Values are given as arithmetic mean ± standard deviation (SD) or number of patients (%)

*^1^ New York Heart Association (NYHA) Functional Classification

*^2^ Myocardial infarction dated back more than 48 hours

*^3^ Minimum, first quartile, median, third quartile, maximum

The study patients had a mean age of approx. 64 years with a ratio of women (n = 17) to men (n = 71) of approximately 1:4. Patients were preobese, with a mean BMI of 27.7, and 7 patients (8%) had no symptoms of angina pectoris.

### Subjective pain intensity and undesired events

**Fig 2:**
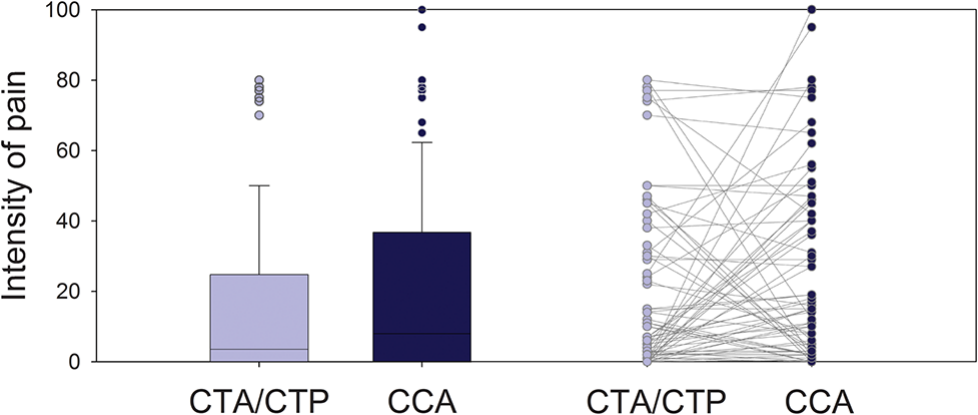
Pain intensity. Boxplot of pain intensities patients reported for CTA/CTP and CCA on the horizontal visual analogue scale (0–100mm) and corresponding intraindividual comparison between the 68 patients who experienced pain during at least one examination; n = 36 pain CCA> CTA/CTP; n = 24 pain CTA/CTP> CCA; n = 8 pain CTA/CTP = CCA; p = 0.121 using the chi-square test.

Twenty patients (23%) reported no pain with either procedure. Seventeen patients (19%) reported subjective pain for only one procedure–five for CTA/CTP and 12 for CCA. Over half of the patients reported pain for both procedures (n = 51, 58%). The averaged pain intensity ratings of all 88 patients did not differ significantly between CTA/CTP (16±23) and CCA (20±26) (p = 0.141; **Fig 2A**). One patient reported maximum pain for CCA during puncture and while the introducer sheath was pulled out; another five patients also reported high pain intensity at the puncture site in the groin (75 to 95 on the VAS, **Fig 2B**). For CTA/CTP, high pain intensity (75 to 80 on the VAS) was reported by five patients (uncomfortable position and cold: n = 3; adenosine-related chest pain: n = 2). CTA was experienced to cause markedly less intense pain (4±9) than CTP (12±18; p<0.001). Chest pain was more common during CTP than during CTA (n = 22; 25% versus n = 4; 5%; p<0.001).

### Patient acceptance

Most patients rated the preparation and information prior to the two diagnostic tests as, good”or, very good”(CTA/CTP 92% and CCA 91%; **Table 2**). There was no significant difference in patient satisfaction with preprocedural preparation between the two tests (p = 0.186). The majority of patients reported no or little concern (CTA/CTP 58% and CCA 53%); again, the difference between CTA/CTP and CCA was not significant (p = 0.097). Six patients stated that they were very concerned prior to the CT examination. Three patients stated they feared possible complications associated with adenosine administration (n = 3). One patient each was concerned because the test was unknown, involved radiation exposure, or because of fear of the diagnosis. Before CCA, 20 patients experienced, intense”(n = 17) or, very intense”(n = 3) concern with most patients giving fear of the risks of an invasive procedure as the reason (n = 8). Other reasons were fear of pain (n = 4) and uncertainty (n = 4). Yet other patients were concerned because they feared the diagnosis (n = 3) or the long time of bedrest after CCA (n = 1).

**Table 2:** Patient Acceptance of CTA/CTP and CCA

	CTA/CTP	CCA	P
Patient preparation	1.5 ± 0.6	1.6 ± 0.7	0.186
Concern	2.1 ± 1.0	2.4 ± 1.2	0.097
Comfort	1.8 ± 0.8	1.8 ± 0.8	0.377
Helplessness	1.5 ± 0.8	1.8 ± 0.9	0.026 *
Overall satisfaction	1.6 ± 0.6	1.8 ± 0.7	0.123

Values are given as arithmetic mean ± SD.

The scale used for preparation and information prior to the test comfort, and overall satisfaction was: 1 = very good, 2 = good, 3 = moderate, 4 = poor, 5 = very poor. Concern was rated as: 1 = no concern, 2 = little, 3 = moderate, 4 = intense, 5 = very intense. Helplessness was rated as: 1 = no helplessness, 2 = little, 3 = moderate, 4 = intense, 5 = very intense.

The ratings for patient preparation, concern, comfort, and overall satisfaction were comparable for CTA/CTP and CCA. Only the ratings for helplessness were significantly different between the two tests; here patients felt significantly less helpless during CTA/CTP than during CCA (* using sign test).

Patients were more concerned about CTP than about CTA (p<0.001). Two patients were concerned because they feared the effects of adenosine administration (n = 2). Fear of an unknown procedure was given as a cause of concern before both CTA and CTP (n = 1 each). Dyspnea was clearly more common during CTP than during CTA (n = 18 or 20% versus n = 2 or 2%; p<0.001).

Comfort during the examinations was rated to be, very good”or, good”(86% CTA/CTP; 91% CCA; P = NS; **Table 2**). No patient considered comfort during CTA/CTP to be “very poor”, whereas one patient experienced comfort during CCA to be “very poor”. The reasons given were invasiveness of the test, the long time of bedrest after the procedure, and the unpleasantness of the compression bandage.

### Patient preference and future examinations

**Fig 3:**
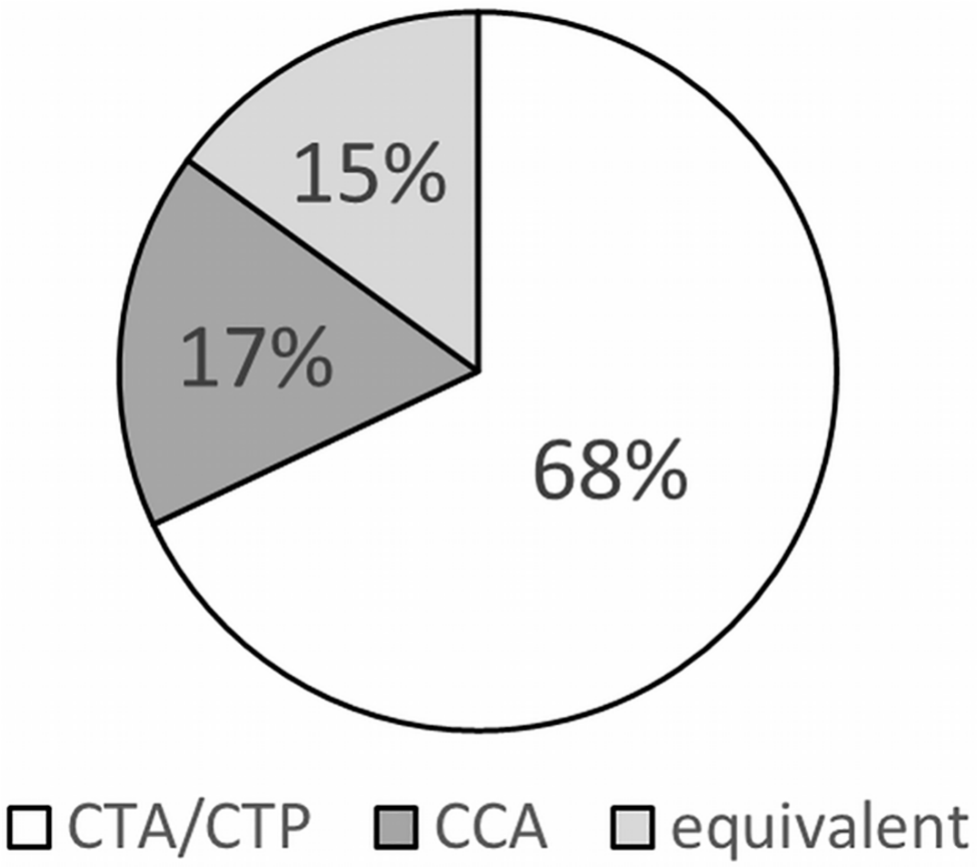
Patient preference. Patient preferences for the different tests; significant preference for CTA/CTP by using the chi-square test.

The majority of patients preferred combined CTA/CTP to CCA (**Fig 3**; p<0.001). The preference for CTA/CTP was significantly higher among the 47 patients who did not have to undergo subsequent coronary revascularization (75%; 35 of 47 patients) than among the patients with subsequent revascularization (61% or 25 of 41; p = 0.002). Most patients were willing to undergo CTA/CTP (71 patients; 81%) and CCA again (75 patients; 85%). There was no difference between the two tests (p = 0.664). Twelve patients each (14%) stated that they did not know whether they would undergo the test again. Very few patients stated that they would not undergo another CTA/CTP (n = 5; 6%) or CCA (n = 1; 1%) examination.

### Open-ended questions

The most common advantage of CTA/CTP mentioned by the patients was the absence of invasiveness with avoidance of the possible risks associated with an invasive procedure. This advantage was mentioned by nearly one third of the responding patients (32%, n = 18; **Table 3**). One quarter of patients mentioned painlessness, and approx. one fifth of patients each appreciated the avoidance of long bedrest after the test and the rapidity of the test compared with CCA. The most common disadvantage, mentioned by 41% of the patients (n = 18), was the inability to perform interventional measures during the test. Several patients mentioned an uncomfortable position and radiation exposure as negative aspects of CTA/CTP. In the evaluation of CCA, more than two thirds of patients (68%, n = 32) appreciated the option of performing an interventional procedure when a coronary stenosis is detected as an advantage. This was the most common advantage mentioned, followed by high diagnostic accuracy of the test from the patient’s perspective. The most common disadvantage of CCA, mentioned by nearly half of the patients (48%, n = 23), was the bedrest. Invasiveness was mentioned as a negative aspect by nearly one third of patients (31%, n = 15). The need for a compression bandage after the test and the occurrence of hematomas were mentioned as other unpleasant aspects of CCA.

**Table 3:** Advantages and Disadvantages of CTA/CTP and CCA Reported by Patients.

	CTA/CTP		CCA	
Advantage	Noninvasiveness	18; 32%	Option to perform intervention	32; 68%
	Painlessness	14; 25%	Diagnostic accuracy	11; 23%
	No bedrest after CT scan	12; 21%	Short duration	5; 11%
	Short duration	11; 19%	Physician contact throughout the test	4; 9%
	Diagnostic accuracy	9; 16%	Painlessness	3; 6%
	Absence of risks	8; 14%	Little discomfort	1; 2%
	Outpatient procedure	5; 9%		
	Assessment of other organs	2; 4%		
	Little physical distress	2; 4%		
	No need for compression bandage	2; 4%		
	Repeatability	1; 2%		
	Inexpensiveness	1; 2%		
	n = 85, given by 55 patients		n = 56, given by 46 patients	
Disadvantage	No treatment possible	18; 41%	Bedrest after the procedure	23; 48%
	Uncomfortable position/little space	9; 20%	Risks of an invasive test	15; 31%
	Radiation exposure	7; 16%	Pressure bandage	11; 23%
	Contrast medium administration	6; 14%	Hematoma	6; 13%
	Chest pain, dyspnea after adenosine administration	4; 9%	Pain	5; 10%
	Limited diagnostic accuracy	3; 7%	Hospitalization	5; 10%
	Long duration of analysis before results are available	1; 2%	Contrast medium administration	3; 6%
			Helplessness	1; 2%
			Little comfort	1; 2%
			Doubt about diagnostic accuracy	1; 2%
	n = 48, given by 38 patients		n = 71, given by 46 patients	

Values are given as numbers of patients and percentages

The most common advantages of CTA/CTP mentioned by patients were noninvasiveness, absence of pain, and the fact that there was no need for bedrest after the test. The major disadvantages were that it was not possible to perform interventional procedures during the test and the uncomfortable position. Advantages patients attributed to CCA were the possibility of directly performing interventional measures and the diagnostic accuracy. Frequently mentioned disadvantages of CCA were the risks associated with the procedure, the long bedrest after the procedure, and the need for a compression bandage.

### Subgroup analysis

The results of subgroup analysis are summarized in **Table 4**. Whether or not revascularization was performed during CCA had no significant effect on the comfort ratings, helplessness experienced during the procedure, or overall satisfaction with CCA. There was also no difference in pain intensities reported by patients with and without adenosine-induced FFR assessment during CCA. Use of the AngioSeal closure system after CCA was not associated with more intense pain than manual compression. Women rated preparation for the examinations as “very good” or “good” in 88% of cases for CTA/CTP and 94% for CCA versus 93% and 90% of men, respectively. No pain during both tests was reported by nearly the same proportions of women and men (53% versus 66%). Mean pain intensity reported for CTA/CTP was not significantly different between women and men. There were also no significant differences with regard to the mean pain intensity experienced during CCA. Overall satisfaction was good and comparable between women and men for CTA/CTP. However, with regard to CCA, overall satisfaction was significantly higher among women (p = 0.002) than among men. CTA/CTP was the procedure of first choice for 59% of women and 70% of men (p = 0.321).

**Table 4:** Subgroup Analyses.

**1.**	**No revascularization**		**Revascularization**		**p**
CCA comfort	1.8	(0.6)	1.9	(0.9)	0.795
CCA helplessness	1.7	(0.9)	1.8	(1.0)	0.946
CCA overall satisfaction	1.7	(0.6)	1.8	(0.9)	0.879
**2.**	**With FFR**		**Without FFR**		**p**
CCA pain	17.0	(15.4)	20.2	(26.6)	0.592
**3.**	**Suture closure system**		**Manual compression**		**p**
CCA pain	19.2	(26.3)	22.1	(24.3)	0.549
**4.**	**Women**		**Men**		**p**
CTA/CTP preparation	1.4	(0.7)	1.6	(0.6)	0.212
CCA preparation	1.4	(0.6)	1.7	(0.7)	0.090
CTA/CTP pain	18.4	(29.4)	15.0	(21.4)	0.558
CCA pain	16.2	(25.7)	20.8	(25.8)	0.271
CTA/CTP overall satisfaction	1.4	(0.6)	1.7	(0.6)	0.117
CCA overall satisfaction	1.3	(0.5)	1.9	(0.8)	0.002

Values are given as arithmetic mean ± standard deviation (SD) or number of patients (%).The scale used for preparation and information prior to the test comfort, and overall satisfaction was: 1 = very good, 2 = good, 3 = moderate, 4 = poor, 5 = very poor. Helplessness was rated as: 1 = no helplessness, 2 = little, 3 = moderate, 4 = intense, 5 = very intense. Pain intensity was assessed on an unmarked 100-mm visual analogue scale. Preferences were given by selecting one of three possible answers: CTA/CTP, CCA, or both equivalent. A total of four subgroup analyses were performed: 1) patients with revascularization versus patients without revascularization during CCA (n = 42 versus n = 46); 2) patients with FFR versus patients without FFR during CCA (n = 8 versus n = 80); 3) patients with suture closure system versus patients with manual compression after CCA (n = 66 versus n = 22); and 4) women versus men (n = 17 versus n = 71). The other subgroup analyses revealed no significant differences

## Discussion

The present study for the first time analyzes patient acceptance of combined CTA/CTP with adenosine administration in comparison to CCA. In our study population of patients with known coronary heart disease and prior stent implantation, CTA/CTP is preferred over CCA. While most single tests were without statistical significant differences, combined CTA/CTP showed slightly higher overall satisfaction, whereas pain intensity values, the degree of concern and helplessness were slightly decreased as compared to CCA. In addition, the freetext analysis may show further reasons for the clear preference of CTA/CTP to CCA: the absence of risks was the most frequently mentioned advantage of CTA/CTP, while the long bedrest time associated with the invasive procedure was the most common disadvantage of the CCA. It is noteworthy that, while the pain experienced during CTA/CTP tended to be less intense, the difference in overall pain was not significantly different between the noninvasive and the invasive test. A closer analysis revealed that intense pain during CT was mainly attributed to myocardial CTP with adenosine administration.

### Comparison with other studies

The coronary CTA shows a high diagnostic accuracy in the detection of significant coronary stenoses [20, 21]. In patients with acute coronary syndrome and a low to intermediate likelihood, CTA is a safe and effacious alternative to the CCA [22], showing the potential for additional diagnostic testing [23]. Beside the diagnostic accuracy, CT may improve the cost-effectiveness in comparison to CCA in patients with an intermediate risk [23, 24]. Thus, CTA may represent an alternative first-step-diagnostic tool, if the necessity of coronary stenting is not likely [23].

The advent of new generations of CT scanners has provided the technical basis for achieving higher temporal resolution or wider detector coverage for myocardial perfusion imaging [25, 26]. Especially in patients with known CAD coronary stents may decrease the diagnostic evaluability. Combined CTA and CTP with administration of adenosine may improve the diagnostic accuracy compared with CTA alone [13, 27–29]. As a result, CTA/CTP has emerged as a new imaging test besides single-photon emission computed tomography, cardiac magnetic resonance imaging, and positron emission tomography [30–32]. An advantage is that myocardial CTP and coronary CTA can be combined and performed in a single session [13, 33–36]. Adenosine has a good safety profile and is widely used for myocardial stress perfusion imaging [37]. In our study, we administered adenosine at a standard dose of 140μg/kg body weight/min over a total of approximately 5 min. Our results are in agreement with an earlier study showing that patients with symptoms of acute coronary syndrome prefer a noninvasive cardiac stress test such as myocardial scintigraphy with drug-induced stress to an invasive procedure [38].

It is noteworthy that the pain intensity reported by our patients was similar for CTA/CTP and CCA. An earlier intraindividual comparison between coronary CTA with CCA and coronary MRA reported less pain and higher comfort for the CT examination without use of adenosine [15]. In agreement with these insights, another study showed coronary CTA to be associated with less pain than CCA [39]. In contrast, our study for the first time shows that patients with coronary stents experience increased pain intensity when undergoing combined CTA and CTP with adenosine administration and that the pain intensity was comparable to that of CCA. The patients attributed relevant complaints mainly to the CTP part of the CT examination. In agreement with the study of Sandgaard et al. [39], pain during CCA was consistently located at the puncture site with possible hematoma occurring at the access site giving rise to additional complaints. Interestingly, as with the study of Sandgaard et al. [39], the majority of our patients, who already had coronary stents, gave CT as the procedure of first choice (85% vs. 68% in our study) although this included CTP. Most studies of noninvasive cardiac imaging investigate diagnostic accuracy, and only a few studies are including the patient`s perspective when assessing new diagnostic tests. The patient’s perspective is pivotal in view of the increasingly wider use of noninvasive cardiac CT and was the focus of this subanalysis of the CARS-320 study.

### Limitations

This study compared combined CTA/CTP with adenosine administration and clinically indicated CCA, which, in the majority of patients, was performed without adenosine administration (80 of 88). Only eight of the study patients underwent clinically indicated CCA with measurement of FFR after adenosine administration. While more recent studies compared CTP and FFR [33, 40], they only did so in terms of diagnostic accuracy and did not assess patient acceptance of the two procedures. In general, the doctors`information prior to the examinations may influence the subjective feelings of the patients. In addition, the level of education might be important. We investigated a relatively small number of patients (n = 90) with a 4:1 ratio of men to women, which limits our data with regard to sex-related differences [41]. On the other hand, our response rate (98%) indicates that our analysis is representative. Because of the rather small patient cohort an intraindividual comparison was performed instead of a randomized trial. This intraindividual comparison however, offers the advantage to evaluate differences between tests in each individual and not only between groups which may differ in characteristics. We evaluated patient acceptance using a validated questionnaire also including open-ended questions that, in general, are subjective and may produce bias. On that account, we performed a descriptive evaluation instead of a statistical analysis for open-ended questions. Moreover, the patients included in our study had at least one CCA before study enrolment and hence already knew this procedure. The earlier experience with CCA might have influenced patient acceptance. All patients underwent the tests in the same order: first CTA/CTP and afterwards CCA. In most patients CT and CCA were performed at the same day which increased the amount of contrast agent and radiation exposure compared to one diagnostic test alone. During the follow-up after 24h and 48h three patients showed creatinine increase of >25%, but in all patients creatinine normalized during further follow-up [13]. See the main study for further details regarding the patients`renal function and the radiation exposure. In addition, the order of the examinations could have introduced a bias for patient satisfaction, but was fixed due to the protocol of our intention-to-diagnose CARS-320 study and could not be changed for the purpose of this secondary analysis because it would have biased the published diagnostic accuracy analysis [13]. Additionally, femoral access was used in all patients during the CCA (initiation of the study 2008), as this was the standard technique in our institution at this time, comparable to the United States which predominantly used a transfemoral approach as described until the year 2011 [42]. Recently, the transradial access for CCA became of more interest, as it showed less local vascular complications at the entry site [43], but the utilization of a transradial approach still varies strongly among countries [44]. According to the study protocol, only patients who already had coronary stents were included. The acceptance of the two tests under investigation by patients without coronary stents should be investigated in further studies.

### Conclusions

In summary, while patients with coronary stents experienced pain intensity of combined CTA/CTP to be similar to that of invasive CCA, they preferred the noninvasive combined CTA/CTP examination over CCA.

## Supporting Information

S1 TREND Checklist TREND checklist. (PDF)

S1 File Patients’ Perception Questionnaire. (DOC)

S1 Protocol Study Protocol. (PDF)

S1 Table Table Questionnaire Patient Acceptance. (XLS)

## References

[1] Stergiopoulos K, Brown DL. Initial coronary stent implantation with medical therapy vs medical therapy alone for stable coronary artery disease: meta-analysis of randomized controlled trials. Archives of internal medicine. 2012;172(4):312–9. Epub 2012/03/01. 10.1001/archinternmed.2011.1484 . 22371919

[2] Palmerini T, Biondi-Zoccai G, Della Riva D, Stettler C, Sangiorgi D, D'Ascenzo F, et al Stent thrombosis with drug-eluting and bare-metal stents: evidence from a comprehensive network meta-analysis. Lancet. 2012;379(9824):1393–402. Epub 2012/03/27. 10.1016/S0140-6736(12)60324-9 . 22445239

[3] Park SJ, Kang SJ, Virmani R, Nakano M, Ueda Y. In-stent neoatherosclerosis: a final common pathway of late stent failure. J Am Coll Cardiol. 2012;59(23):2051–7. Epub 2012/06/02. 10.1016/j.jacc.2011.10.909 . 22651862

[4] Qaseem A, Fihn SD, Williams S, Dallas P, Owens DK, Shekelle P. Diagnosis of stable ischemic heart disease: summary of a clinical practice guideline from the American College of Physicians/American College of Cardiology Foundation/American Heart Association/American Association for Thoracic Surgery/Preventive Cardiovascular Nurses Association/Society of Thoracic Surgeons. Ann Intern Med. 2012;157(10):729–34. Epub 2012/11/21. 10.7326/0003-4819-157-10-201211200-00010 . 23165664

[5] Schuetz GM, Zacharopoulou NM, Schlattmann P, Dewey M. Meta-analysis: noninvasive coronary angiography using computed tomography versus magnetic resonance imaging. Ann Intern Med. 2010;152(3):167–77. 10.7326/0003-4819-152-3-201002020-00008 . 20124233

[6] Choi JH, Min JK, Labounty TM, Lin FY, Mendoza DD, Shin DH, et al Intracoronary transluminal attenuation gradient in coronary CT angiography for determining coronary artery stenosis. JACC Cardiovascular imaging. 2011;4(11):1149–57. Epub 2011/11/19. 10.1016/j.jcmg.2011.09.006 . 22093264

[7] Sun Z, Davidson R, Lin CH. Multi-detector row CT angiography in the assessment of coronary in-stent restenosis: a systematic review. Eur J Radiol. 2009;69(3):489–95. Epub 2007/12/29. 10.1016/j.ejrad.2007.11.030 . 18162351

[8] Ko BS, Cameron JD, Leung M, Meredith IT, Leong DP, Antonis PR, et al Combined CT coronary angiography and stress myocardial perfusion imaging for hemodynamically significant stenoses in patients with suspected coronary artery disease: a comparison with fractional flow reserve. JACC Cardiovascular imaging. 2012;5(11):1097–111. Epub 2012/11/17. 10.1016/j.jcmg.2012.09.004 . 23153909

[9] Vavere AL, Simon GG, George RT, Rochitte CE, Arai AE, Miller JM, et al Diagnostic performance of combined noninvasive coronary angiography and myocardial perfusion imaging using 320 row detector computed tomography: design and implementation of the CORE320 multicenter, multinational diagnostic study. J Cardiovasc Comput Tomogr. 2011;5(6):370–81. Epub 2011/12/08. 10.1016/j.jcct.2011.11.001 . 22146496 PMC3828643

[10] Tashakkor AY, Nicolaou S, Leipsic J, Mancini GB. The emerging role of cardiac computed tomography for the assessment of coronary perfusion: a systematic review and meta-analysis. Can J Cardiol. 2012;28(4):413–22. 10.1016/j.cjca.2012.02.010 . 22542048

[11] Bettencourt N, Ferreira ND, Leite D, Carvalho M, Ferreira Wda S, Schuster A, et al CAD detection in patients with intermediate-high pre-test probability: low-dose CT delayed enhancement detects ischemic myocardial scar with moderate accuracy but does not improve performance of a stress-rest CT perfusion protocol. JACC Cardiovascular imaging. 2013;6(10):1062–71. Epub 2013/09/10. 10.1016/j.jcmg.2013.04.013 . 24011773

[12] Keirns CC, Goold SD. Patient-centered care and preference-sensitive decision making. JAMA. 2009;302(16):1805–6. 10.1001/jama.2009.1550 . 19861674

[13] Rief M, Zimmermann E, Stenzel F, Martus P, Stangl K, Greupner J, et al CT Angiography and Myocardial CT Perfusion in Patients with Coronary Stents: Prospective Intraindividual Comparison with Conventional Coronary Angiography. J Am Coll Cardiol. 2013 10.1016/j.jacc.2013.03.088 . 23792193

[14] Des Jarlais DC, Lyles C, Crepaz N, Group T. Improving the reporting quality of nonrandomized evaluations of behavioral and public health interventions: the TREND statement. Am J Public Health. 2004;94(3):361–6. 14998794 10.2105/ajph.94.3.361 PMC1448256

[15] Schönenberger E, Schnapauff D, Teige F, Laule M, Hamm B, Dewey M. Patient acceptance of noninvasive and invasive coronary angiography. PLoS One. 2007;2(2):e246 10.1371/journal.pone.0000246 17327910 PMC1796945

[16] Rief M, Stenzel F, Kranz A, Schlattmann P, Dewey M. Time efficiency and diagnostic accuracy of new automated myocardial perfusion analysis software in 320-row CT cardiac imaging. Korean J Radiol. 2013;14(1):21–9. 10.3348/kjr.2013.14.1.21 23323027 PMC3542299

[17] Miller JM, Dewey M, Vavere AL, Rochitte CE, Niinuma H, Arbab-Zadeh A, et al Coronary CT angiography using 64 detector rows: methods and design of the multi-centre trial CORE-64. Eur Radiol. 2009;19(4):816–28. 10.1007/s00330-008-1203-7 18998142 PMC3289939

[18] Wijns W, Kolh P, Danchin N, Di Mario C, Falk V, Folliguet T, et al Guidelines on myocardial revascularization. Eur Heart J. 2010;31(20):2501–55. Epub 2010/08/31. 10.1093/eurheartj/ehq277 . 20802248

[19] Budoff MJ, Achenbach S, Blumenthal RS, Carr JJ, Goldin JG, Greenland P, et al Assessment of coronary artery disease by cardiac computed tomography: a scientific statement from the American Heart Association Committee on Cardiovascular Imaging and Intervention, Council on Cardiovascular Radiology and Intervention, and Committee on Cardiac Imaging, Council on Clinical Cardiology. Circulation. 2006;114(16):1761–91. 10.1161/CIRCULATIONAHA.106.178458 . 17015792

[20] Dewey M, Zimmermann E, Deissenrieder F, Laule M, Dübel HP, Schlattmann P, et al Noninvasive coronary angiography by 320-row computed tomography with lower radiation exposure and maintained diagnostic accuracy: comparison of results with cardiac catheterization in a head-to-head pilot investigation. Circulation. 2009;120(10):867–75. 10.1161/CIRCULATIONAHA.109.859280 . 19704093

[21] Pelliccia F, Pasceri V, Evangelista A, Pergolini A, Barillà F, Viceconte N, et al Diagnostic accuracy of 320-row computed tomography as compared with invasive coronary angiography in unselected, consecutive patients with suspected coronary artery disease. Int J Cardiovasc Imaging. 2012 10.1007/s10554-012-0095-4 . 22806317

[22] Kim HR, Yoo SM, Rho JY, Lee HY, White CS. MDCT evaluation of atherosclerotic coronary artery disease: what should radiologists know? Int J Cardiovasc Imaging. 2014;30 Suppl 1:1–11. 10.1007/s10554-014-0411-2 . 24687407

[23] D'Ascenzo F, Cerrato E, Biondi-Zoccai G, Omedè P, Sciuto F, Presutti DG, et al Coronary computed tomographic angiography for detection of coronary artery disease in patients presenting to the emergency department with chest pain: a meta-analysis of randomized clinical trials. Eur Heart J Cardiovasc Imaging. 2013;14(8):782–9. 10.1093/ehjci/jes287 . 23221314

[24] Darlington M, Gueret P, Laissy JP, Pierucci AF, Maoulida H, Quelen C, et al Cost-effectiveness of computed tomography coronary angiography versus conventional invasive coronary angiography. Eur J Health Econ. 2014 10.1007/s10198-014-0616-2 . 24990117

[25] Bamberg F, Klotz E, Flohr T, Becker A, Becker CR, Schmidt B, et al Dynamic myocardial stress perfusion imaging using fast dual-source CT with alternating table positions: initial experience. Eur Radiol. 2010;20(5):1168–73. Epub 2010/03/25. 10.1007/s00330-010-1715-9 . 20333388

[26] Dewey M, Zimmermann E, Deissenrieder F, Laule M, Dubel HP, Schlattmann P, et al Noninvasive coronary angiography by 320-row computed tomography with lower radiation exposure and maintained diagnostic accuracy: comparison of results with cardiac catheterization in a head-to-head pilot investigation. Circulation. 2009;120(10):867–75. 10.1161/CIRCULATIONAHA.109.859280 19704093

[27] Hosokawa K, Kurata A, Kido T, Shikata F, Imagawa H, Kawachi K, et al Transmural perfusion gradient in adenosine triphosphate stress myocardial perfusion computed tomography. Circ J. 2011;75(8):1905–12. . 21697608 10.1253/circj.cj-10-1144

[28] Schuijf JD, Wijns W, Jukema JW, Atsma DE, de Roos A, Lamb HJ, et al Relationship between noninvasive coronary angiography with multi-slice computed tomography and myocardial perfusion imaging. J Am Coll Cardiol. 2006;48(12):2508–14. 10.1016/j.jacc.2006.05.080 . 17174190

[29] Ko SM, Choi JW, Hwang HK, Song MG, Shin JK, Chee HK. Diagnostic performance of combined noninvasive anatomic and functional assessment with dual-source CT and adenosine-induced stress dual-energy CT for detection of significant coronary stenosis. AJR Am J Roentgenol. 2012;198(3):512–20. 10.2214/AJR.11.7029 . 22357990

[30] Kim YH, Ahn JM, Park DW, Song HG, Lee JY, Kim WJ, et al Impact of ischemia-guided revascularization with myocardial perfusion imaging for patients with multivessel coronary disease. J Am Coll Cardiol. 2012;60(3):181–90. Epub 2012/07/14. 10.1016/j.jacc.2012.02.061 . 22789882

[31] Jaarsma C, Leiner T, Bekkers SC, Crijns HJ, Wildberger JE, Nagel E, et al Diagnostic performance of noninvasive myocardial perfusion imaging using single-photon emission computed tomography, cardiac magnetic resonance, and positron emission tomography imaging for the detection of obstructive coronary artery disease: a meta-analysis. J Am Coll Cardiol. 2012;59(19):1719–28. Epub 2012/05/05. 10.1016/j.jacc.2011.12.040 . 22554604

[32] Dorbala S, Di Carli MF, Beanlands RS, Merhige ME, Williams BA, Veledar E, et al Prognostic value of stress myocardial perfusion positron emission tomography: results from a multicenter observational registry. J Am Coll Cardiol. 2013;61(2):176–84. Epub 2012/12/12. 10.1016/j.jacc.2012.09.043 23219297 PMC3549438

[33] Bettencourt N, Chiribiri A, Schuster A, Ferreira N, Sampaio F, Pires-Morais G, et al Direct comparison of cardiac magnetic resonance and multidetector computed tomography stress-rest perfusion imaging for detection of coronary artery disease. J Am Coll Cardiol. 2013;61(10):1099–107. Epub 2013/02/05. 10.1016/j.jacc.2012.12.020 . 23375929

[34] Bamberg F, Becker A, Schwarz F, Marcus RP, Greif M, von Ziegler F, et al Detection of hemodynamically significant coronary artery stenosis: incremental diagnostic value of dynamic CT-based myocardial perfusion imaging. Radiology. 2011;260(3):689–98. Epub 2011/08/19. 10.1148/radiol.11110638 . 21846761

[35] Blankstein R, Shturman LD, Rogers IS, Rocha-Filho JA, Okada DR, Sarwar A, et al Adenosine-induced stress myocardial perfusion imaging using dual-source cardiac computed tomography. J Am Coll Cardiol. 2009;54(12):1072–84. Epub 2009/09/12. 10.1016/j.jacc.2009.06.014 . 19744616

[36] Feuchtner G, Goetti R, Plass A, Wieser M, Scheffel H, Wyss C, et al Adenosine stress high-pitch 128-slice dual-source myocardial computed tomography perfusion for imaging of reversible myocardial ischemia: comparison with magnetic resonance imaging. Circulation Cardiovascular imaging. 2011;4(5):540–9. Epub 2011/08/25. 10.1161/CIRCIMAGING.110.961250 . 21862731

[37] Cerqueira MD, Verani MS, Schwaiger M, Heo J, Iskandrian AS. Safety profile of adenosine stress perfusion imaging: results from the Adenoscan Multicenter Trial Registry. J Am Coll Cardiol. 1994;23(2):384–9. Epub 1994/02/01. . 8294691 10.1016/0735-1097(94)90424-3

[38] Mumma BE, Baumann BM, Diercks DB, Takakuwa KM, Campbell CF, Shofer FS, et al Sex bias in cardiovascular testing: the contribution of patient preference. Ann Emerg Med. 2011;57(6):551–60.e4. 10.1016/j.annemergmed.2010.09.026 . 21146255

[39] Sandgaard NC, Diederichsen AC, Petersen H, Hoilund-Carlsen PF, Mickley H. Patients' views of cardiac computed tomography angiography compared with conventional coronary angiography. Journal of thoracic imaging. 2012;27(1):36–9. Epub 2011/03/26. 10.1097/RTI.0b013e3182108091 . 21436742

[40] Ko BS, Cameron JD, Meredith IT, Leung M, Antonis PR, Nasis A, et al Computed tomography stress myocardial perfusion imaging in patients considered for revascularization: a comparison with fractional flow reserve. Eur Heart J. 2012;33(1):67–77. Epub 2011/08/04. 10.1093/eurheartj/ehr268 . 21810860

[41] Schuetz GM, Schlattmann P, Achenbach S, Budoff M, Garcia MJ, Roehle R, et al Individual patient data meta-analysis for the clinical assessment of coronary computed tomography angiography: protocol of the Collaborative Meta-Analysis of Cardiac CT (CoMe-CCT). Systematic reviews. 2013;2(1):13 Epub 2013/02/19. 10.1186/2046-4053-2-13 23414575 PMC3576350

[42] Dehmer GJ, Weaver D, Roe MT, Milford-Beland S, Fitzgerald S, Hermann A, et al A contemporary view of diagnostic cardiac catheterization and percutaneous coronary intervention in the United States: a report from the CathPCI Registry of the National Cardiovascular Data Registry, 2010 through June 2011. J Am Coll Cardiol. 2012;60(20):2017–31. Epub 2012/10/23. 10.1016/j.jacc.2012.08.966 . 23083784

[43] Jolly SS, Yusuf S, Cairns J, Niemela K, Xavier D, Widimsky P, et al Radial versus femoral access for coronary angiography and intervention in patients with acute coronary syndromes (RIVAL): a randomised, parallel group, multicentre trial. Lancet. 2011;377(9775):1409–20. Epub 2011/04/08. 10.1016/S0140-6736(11)60404-2 . 21470671

[44] Caputo RP, Tremmel JA, Rao S, Gilchrist IC, Pyne C, Pancholy S, et al Transradial arterial access for coronary and peripheral procedures: executive summary by the Transradial Committee of the SCAI. Catheterization and cardiovascular interventions: official journal of the Society for Cardiac Angiography & Interventions. 2011;78(6):823–39. Epub 2011/05/06. 10.1002/ccd.23052 . 21544927

