# Supplementary material for: Acceptance of Combined Coronary CT Angiography and Myocardial CT Perfusion versus Conventional Coronary Angiography in Patients with Coronary Stents—Intraindividual Comparison
Source: PLoS One. 2015 Sep 1;10(9):e0136737. doi: 10.1371/journal.pone.0136737 (PMC4556695; doi:10.1371/journal.pone.0136737)
Supplement: S1 File — (DOC) [file pone.0136737.s002.doc]

**Patients’ Perception Questionnaire** Patient´s name: …………………

In the following we would like to introduce you to our patients´ perception questionnaire, which we wish you to complete as part of the “CARS-320 study”. The aim of the questions is to ask your personal feelings about the diagnostic tests you had and your satisfaction with each test. We reassure you that your answers will in no way affect your further treatment, no matter how critical or positive your answers are.

**CTA and CTP**

1. Please rate the preparation and information prior to this test.

Very good 🔾 Good 🔾 Moderate 🔾 Poor 🔾 Very poor🔾

2. Please rate your degree of concern prior to this test.

No concern 🔾 Little 🔾 Moderate 🔾 Intense 🔾 Very intense🔾

In case you were concerned: **Why** were you concerned? ……………………………..

3. Please rate the comfort of this test.

Very good 🔾 Good 🔾 Moderate 🔾 Poor 🔾 Very poor🔾

4. Please rate your degree of helplessness during this test.

No helplessness 🔾 Little 🔾 Moderate 🔾 Intense 🔾 Very intense🔾

5. Please rate your pain during and immediately after this test on the following scale

(measure from left end of scale). The test was divided into two parts. Please mark with colors the amount of pain caused by the rest and stress examination as compared to the whole test.

*No pain Maximum pain*

CTA (rest examination) green / CTP (stress examination) red

6. Did complications occur during or after the test? Yes 🔾 No 🔾

If yes, **what kind** of complications did you have? ..........................................................

7. Would you be willing to undergo this test again?

Yes 🔾 No 🔾 Don´t know 🔾

8. Please rate your overall satisfaction with this test.

Very Good 🔾 Good 🔾 Moderate 🔾 Poor 🔾 Very poor 🔾

**Conventional Coronary Angiography**

1. Please rate the preparation and information prior to this test.

Very good 🔾 Good 🔾 Moderate 🔾 Poor 🔾 Very poor🔾

2. Please rate your degree of concern prior to this test.

No concern 🔾 Little 🔾 Moderate 🔾 Intense 🔾 Very intense🔾

In case you were concerned: Why were you concerned? ……………………………..

3. Please rate the comfort of this test.

Very good 🔾 Good 🔾 Moderate 🔾 Poor 🔾 Very poor🔾

4. Please rate your degree of helplessness during this test.

No helplessness 🔾 Little 🔾 Moderate 🔾 Intense 🔾 Very intense🔾

5. Please rate your pain during and immediately after this test on the following scale

(measure from left end of scale).

*No pain Maximum pain*

6. Did complications occur during or after the test? Yes 🔾 No 🔾

If yes, what kind of complications did you have? ..........................................................

7. Would you be willing to undergo this test again?

Yes 🔾 No 🔾 Don´t know 🔾

8. Please rate your overall satisfaction with this test.

Very good 🔾 Good 🔾 Moderate 🔾 Poor 🔾 Very poor 🔾

**Summary**

1. Which test would you prefer for future **diagnostic imaging**? Please name your favorite test first.

1. ……..………..…… 2. ……..………..……

2. Please describe other details that are important to you regarding the two tests and how you feel about them on the following lines. In the table below, you can list what you personally think are important advantages and disadvantages of each test.

……………………………………………………………………………………………………………..

……………………………………………………………………………………………………………..

……………………………………………………………………………………………………………..

|  | Advantages | Disadvantages |
| --- | --- | --- |
| CT |  |  |
| Coronary catheter |  |  |

Your cardiac CT scan was divided into two parts: first the cardiac examination at rest (“rest examination”) and afterwards the examination with pharmacological stress to measure blood flow in the heart muscle using adenosine (“stress examination”). Please answer the following questions so we can find out how stressful each part of the CT scan is experienced by you and other patients.

**CTA (rest examination):**

1. Please rate your degree of concern prior to this test.

No concern 🔾 Little 🔾 Moderate 🔾 Intense 🔾 Very intense🔾

In case you were concerned: Why were you concerned? ……………………………..

2. Did you experience shortness of breath, chest pain or pressure on the chest during the rest examination?

Yes 🔾 No 🔾

If yes, what kind of distress did you experience? _____________________________________________

**CTP (stress examination):**

1. Please rate your degree of concern prior to this test.

No concern 🔾 Little 🔾 Moderate 🔾 Intense 🔾 Very intense🔾

In case you were concerned: Why were you concerned? ……………………………..

2. Did you experience shortness of breath, chest pain or pressure on the chest during the rest examination?

Yes 🔾 No 🔾

If yes, what kind of distress did you experience? _____________________________________________

**Repeat CT examination**

1. Would you be willing to undergo a CTP examination with adenosine (“stress examination”) again?

Yes 🔾 No 🔾 Don´t know🔾

If no, why not? ……………………………..

2. Would you be willing to undergo a CTA examination without adenosine (“rest examination”) again?

Yes 🔾 No 🔾 Don´t know🔾

If no, why not? ……………………………..

Thank you very much for taking the time to complete this questionnaire
